# Buffalo Medical College

**Published:** 1849-09

**Authors:** 


*Buffalo Medical College.*—To obviate any erroneous impressions, we take occasion again to state, that the completion of the new college edifice is in rapid progress, and that it will be in readiness for occupancy by the commencement of the next session.

The preliminary course in this institution, as will be seen by the advertisement, will commence on the 10th proximo. Daily lectures will be given in the new college by the Professors of Surgery, Chemistry, and Medicine ; clinical lectures on cases at the adjoining hospital of the Sisters of Charity, and dissections prosecuted under the direction of Prof. Ford. The regular term commences on the 7th of November, and continues sixteen weeks.

